# Prospective Study of the Diagnostic Accuracy of the In Vivo Laser Scanning Confocal Microscope for Severe Microbial Keratitis

**DOI:** 10.1016/j.ophtha.2016.07.009

**Published:** 2016-11

**Authors:** Jaya D. Chidambaram, Namperumalsamy V. Prajna, Natasha L. Larke, Srikanthi Palepu, Shruti Lanjewar, Manisha Shah, Shanmugam Elakkiya, Prajna Lalitha, Nicole Carnt, Minna H. Vesaluoma, Melanie Mason, Scott Hau, Matthew J. Burton

**Affiliations:** 1 London School of Hygiene and Tropical Medicine, London, United Kingdom; 2 Aravind Eye Hospital, Madurai, Tamil Nadu, India; 3 Aravind Medical Research Foundation, Madurai, Tamil Nadu, India; 4 Moorfields Eye Hospital, London, United Kingdom

**Keywords:** AK, *Acanthamoeba* keratitis, BA, blood agar, CI, confidence interval, IVCM, in vivo confocal microscopy, MK, microbial keratitis, NPV, negative predictive value, PDA, potato dextrose agar, PPV, positive predictive value

## Abstract

**Purpose:**

To determine the diagnostic accuracy of in vivo confocal microscopy (IVCM) for moderate to severe microbial keratitis (MK).

**Design:**

Double-masked prospective cohort study.

**Participants:**

Consecutive patients presenting to Aravind Eye Hospital, Madurai, India, between February 2012 and February 2013 with MK (diameter ≥3 mm, excluding descemetocele, perforation, or herpetic keratitis).

**Methods:**

Following examination, the corneal ulcer was scanned by IVCM (HRT3/RCM, Heidelberg Engineering, Heidelberg, Germany). Images were graded for the presence or absence of fungal hyphae or *Acanthamoeba* cysts by the confocal microscopist who performed the scan (masked to microbial diagnosis) and 4 other experienced confocal graders (masked to clinical features and microbiology). The regrading of the shuffled image set was performed by 3 graders, 3 weeks later. Corneal-scrape samples were collected for microscopy and culture.

**Main Outcome Measures:**

The main outcome measures were sensitivity, specificity, and positive and negative predictive values of IVCM compared with those of a reference standard of positive culture or light microscopy. Sensitivities and specificities for multiple graders were pooled and 95% confidence intervals calculated using a bivariate random-effects regression model.

**Results:**

The study enrolled 239 patients with MK. Fungal infection was detected in 176 (74%) and *Acanthamoeba* in 17 (7%) by microbiological methods. IVCM had an overall pooled (5 graders) sensitivity of 85.7% (95% confidence interval [CI]: 82.2%–88.6%) and pooled specificity of 81.4% (95% CI: 76.0%–85.9%) for fungal filament detection. For *Acanthamoeba*, the pooled sensitivity was 88.2% (95% CI: 76.2%–94.6%) and pooled specificity was 98.2% (95% CI: 94.9%–99.3%). Intergrader agreement was good: κ was 0.88 for definite fungus; κ was 0.72 for definite *Acanthamoeba*. Intragrader repeatability was high for both definite fungus (κ: 0.88–0.95) and definite *Acanthamoeba* classification (κ: 0.63–0.90). IVCM images from 11 patients were considered by all 5 graders to have a specific organism present (10 fungus, 1 *Acanthamoeba*) but had negative results via culture and light microscopy.

**Conclusions:**

Laser scanning IVCM performed with experienced confocal graders has high sensitivity, specificity, and test reproducibility for detecting fungal filaments and *Acanthamoeba* cysts in moderate to large corneal ulcers in India. This imaging modality was particularly useful for detecting organisms in deep ulcers in which culture and light microscopy results were negative.

Severe microbial keratitis (MK) is an important cause of blindness worldwide.^1^ In recent years, outbreaks of fungal and *Acanthamoeba* keratitis (AK) have brought to light the complexity of identifying a causative organism in these infections.^2^ Although experienced cornea specialists can correctly identify fungal from bacterial keratitis based on clinical features alone in ≤66% of cases,^3^ larger ulcers may present a diagnostic challenge, as tissue destruction may obscure classical features.^2^ In these cases, microbiological techniques such as culture and light microscopy can aid in diagnosis but they do not offer a high diagnostic accuracy. Culture-positivity rates in MK vary widely, from 40% to 73% in different settings, most likely because of the small size of corneal-scrape samples, prior antimicrobial treatment inhibiting microbial growth, and the fastidious nature of some organisms requiring special growth media (e.g., fungi and *Acanthamoeba*).^4^, ^5^, ^6^, ^7^ Direct visualization of fungal filaments or *Acanthamoeba* cysts in corneal scrapings using light microscopy can give a higher detection rate when compared with culture alone,^8^ but it relies on the availability of trained, experienced observers who may not be present in some health care settings.

In vivo confocal microscopy (IVCM) is a noninvasive imaging technique that allows direct visualization of pathogens within the patient's cornea.^9^ The 2 imaging modalities in current clinical use are the scanning slit IVCM (ConfoScan, Nidek Technologies, Fremont, CA) and the laser scanning IVCM (HRT3 with Rostock Corneal Module [RCM], Heidelberg Engineering, Heidelberg, Germany). The ConfoScan has a resolution of 1 micron laterally and up to 24 microns axially; the HRT3/RCM also has a lateral resolution of 1 micron but higher axial resolution of 7.6 microns.^10^ Although many have reported the ability of both of these confocal microscopes to detect fungal filaments and *Acanthamoeba* cysts in human MK in vivo (summarized in Labbe et al^9^), only 2 studies have prospectively assessed the diagnostic accuracy of IVCM compared with standard microbiological techniques of culture with or without light microscopy.^11^, ^12^ Kanavi et al found that with a single IVCM grader, the ConfoScan 3.0 IVCM had a sensitivity of 100% for detection of *Acanthamoeba* and specificity of 84% compared with culture as the reference standard. For fungal filaments, the sensitivity was also high (94%) but the specificity lower (78%). The authors do not state whether the IVCM grader was masked to data from a clinical assessment of the patient. Vaddavalli et al also used the ConfoScan 3.0 with 2 IVCM graders who were masked to both the microbiological diagnosis and clinical assessment.^12^ They found a sensitivity of 80% and specificity of 100% for the detection of *Acanthamoeba* cysts. For fungal filament detection, they found a sensitivity of 89.2% and specificity of 92.7%. In addition, a good interobserver agreement (κ 0.6) was found for the 2 graders. Hau et al have previously demonstrated that the diagnostic accuracy of IVCM for the diagnosis of MK is also affected by the experience of the IVCM grader.^13^ As such, there is a need to determine the extent of variability between graders in the clinical setting. Resolution of the IVCM imaging system may also affect the ability of graders to detect pathogens, but to date there have been no formal prospective studies using the higher resolution HRT3 IVCM in the detection of MK.

In this study, we aimed to determine the diagnostic accuracy of HRT3 IVCM in moderate to severe MK in South India using 5 experienced confocal graders (masked to microbiological diagnosis). We also assessed intergrader and intragrader agreement.

## Methods

This study was approved by the institutional review board of Aravind Eye Hospital, Tamil Nadu, India; the Indian Council for Medical Research; and the Ethics Committee of the London School of Hygiene and Tropical Medicine. Prior to enrollment in the study, all patients gave written informed consent; study participants who were illiterate gave informed consent with a witnessed thumbprint on the study consent form, as approved by the above ethics committees. This study adhered to the tenets of the Declaration of Helsinki and was conducted in accordance with the Standards for Reporting of Diagnostic Accuracy studies (STARD)^14^—see the STARD checklist in Supplementary Table S1, available at aaojournal.org.

### Study Participants

This study was based in the Cornea Clinic at Aravind Eye Hospital, Madurai, Tamil Nadu, India. Consecutive patients presenting to the clinic between February 2012 and February 2013 were assessed for eligibility and prospectively enrolled into the study if they were found eligible. The inclusion criteria were age ≥18 years and the presence of a large corneal ulcer, defined as a stromal infiltrate ≥3 mm at the longest diameter, with an overlying epithelial defect and signs of acute inflammation. All eligible patients underwent slit lamp examination by an ophthalmologist (cornea specialist), and relevant clinical history and examination findings were recorded in the standardized study form. We excluded any patients with a descemetocele or >80% corneal thinning in the affected eye as assessed on slit lamp examination (i.e., in whom we could not safely applanate the IVCM onto the cornea for imaging), those considered to have herpetic stromal keratitis on clinical grounds (i.e., either a prior history of the disease or the presence of clinical features associated with herpetic disease), or if Snellen visual acuity was worse than 6/60 in the unaffected eye.

### IVCM Imaging

The affected eye was anesthetized using 0.5% proparacaine eye drops (Aurocaine, Aurolab, Madurai, India), and volume scans of the corneal ulcer were obtained using the HRT3 IVCM (Heidelberg Engineering, Heidelberg, Germany) with RCM (63× magnification objective lens, Nikon, Tokyo, Japan), by an ophthalmologist trained in performing IVCM and following a standard procedure described elsewhere.^13^ Briefly, volume scans were obtained in the center of the ulcer, and at the 12-, 3-, 6-, and 9-o'clock positions of the peripheral ulcer margins. Volume scans were taken from the surface of the ulcer and manually refocused several times to take progressively deeper overlapping scan sets covering as much of the full depth of the ulcer as possible.

Immediately after IVCM imaging, the patient underwent scraping of the ulcer base and leading margin for microscopy and culture. The confocal microscopist who performed IVCM imaging was masked to the microbiological diagnosis but had examined the ulcer at the slit lamp prior to performing IVCM. At the time of image acquisition, this grader (grader 5) was asked to grade the IVCM images for the presence or absence of fungal filaments or *Acanthamoeba* cysts; if the grader was suspicious but not confidently certain of a presence, then the image was graded as the possible presence of filaments or cysts.

### Microbiological Diagnosis

Immediately after IVCM had been performed and grading recorded, the base and leading edge of the corneal ulcer were scraped using a flame-sterilized Kimura spatula. Scrapings were immediately placed onto 2 glass slides for light microscopy and on agar plates for culture: blood agar (BA), potato dextrose agar (PDA), and nonnutrient agar seeded with *Escherichia coli* in the laboratory if AK was clinically suspected. Standard microbiological methods were followed to detect any pathogen.^15^ In brief, slides were stained with 10% potassium hydroxide or gram or Giemsa stain to aid in the visualization of fungal filaments, bacteria, or *Acanthamoeba* cysts, respectively; agar plates were incubated at 37° C for 2 days for BA or at 27° C for 7 days for PDA and were assessed daily for organism growth. A culture was classified as positive if any of the following criteria were satisfied: (a) growth of the same species of bacteria or fungus on at least 2 solid media, or (b) semiconfluent growth at the site of inoculation in 1 solid medium of an organism that, for bacteria, was the same as the organism identified with gram stain on microscopy. Organism speciation was performed using standard laboratory methods.^15^ For fungal identification, spores were stained with lactophenol cotton blue and speciated by the morphological appearance of the colony, hyphae, and spores.^16^

### IVCM Grading

Patient-identifying data were removed from all IVCM scans, and images were arranged in a random order for each observer to assess. At Moorfields Eye Hospital, our confocal graders assessed all scans of all recruited patients and graded for the definite presence, definite absence, or possible presence of fungal filaments or *Acanthamoeba* cysts as described above for grader 5. All graders had varying experience of performing IVCM and grading confocal images for MK, ranging from 6 years (graders 1 and 2; grader 2 with an additional 2 years of general IVCM experience), to 3.5 years (grader 3), to 2 years (graders 4 and 5, specifically with IVCM MK imaging experience). All graders were masked to the microbiological diagnosis. Graders 1 to 4 were masked to the clinical appearance of the ulcer. Grading data were directly entered into a Microsoft Access 2010 database. To measure intragrader agreement, all image sets were allocated a new random study number and shuffled into a new order. Three graders were able to repeat the grading process at least 3 weeks after the first grading session.

### Reference Standard

For the purposes of this study, the reference for diagnosis of fungus was a positive culture or (if the culture was negative) the presence of fungal hyphae on light microscopy, as has been used in previous studies.^17^ Similarly, the reference for *Acanthamoeba* was a positive culture or presence of *Acanthamoeba* cysts on light microscopy; this approach has previously been shown to increase diagnostic accuracy for *Acanthamoeba* detection compared with the use of culture alone.^18^ One experienced microbiologist performed the culture and light microscopy interpretation and was masked to the IVCM images and grading but had access to a limited clinical history that was available on the microbiology test request form.

### Statistical Methods

All statistical analyses were performed in Stata version 12.1 (StataCorp, College Station, TX). Sample size was estimated as n = 200 based on a fungal keratitis prevalence estimate of 50%, aiming for a sensitivity of 85%, and with marginal error of 7%, in accordance with Hajjan-Tilaki et al.^19^ Statistical significance of between-group differences in demographic or clinical features was assessed using the Kruskal–Wallis test and chi-square test for proportions. Sensitivity (i.e., ratio of true-positives/true-positives plus false-negatives), specificity (i.e., ratio of true-negatives/true-negatives plus false-positives), positive predictive value, and negative predictive value were calculated using definite fungus or definite *Acanthamoeba* grades for the primary analysis. The primary outcome measure was the pooled sensitivity and specificity of the 5 graders, calculated along with 95% confidence intervals using a bivariate random-effects regression model that accounts for the correlation between the 2 measures (*metandi* and *midas* commands in Stata).^20^, ^21^, ^22^ This is likely to be a conservative estimate because it accounts for the various levels of experience of the graders and only 1 grader takes into account the clinical features of the ulcer. Comparison of regraded outcomes with initial grades was performed using the κ score to calculate intragrader agreement (to assess reproducibility). A κ score was also calculated for intergrader agreement (to assess reliability) for cases graded with certainty as definite fungus*/Acanthamoeba* or no organism seen. The κ scores were interpreted as follows: ≤0.20 *no* agreement; 0.2 to 0.39 minimal agreement; 0.40 to 0.59 weak agreement; 0.60 to 0.79 moderate agreement; 0.80 to 0.90 strong agreement; >0.90 almost perfect agreement.^23^

## Results

### Study Participants

A total of 254 patients were assessed for study eligibility between February 2012 and February 2013, of whom 13 patients were excluded for history of herpetic keratitis (n = 1) or presence of >80% corneal thinning (n = 12). Two patients were also excluded as we were unable to perform diagnostic tests for them: no culture or light microscopy performed (n = 1, deep stromal abscess), or total ulcer with no clear cornea to scan with IVCM (n = 1)—see Supplementary Figure S1 for STARD patient flow diagram, available at www.aaojournal.org. A total of 3163 volume scans were obtained with a mean 13 volume scans per patient (range 3–42 scans). A few patients (n = 4) were unable to cooperate for the full IVCM imaging protocol, and so we were only able to image part of the ulcer—these patients were not excluded. No adverse events were noted from either performing IVCM imaging or corneal scraping for culture or light microscopy.

Sociodemographic features of the final participants are shown in Table 1. Compared with all others, AK patients had a higher frequency of ring infiltrate (88% in AK vs. 31% all others, *P* < 0.0001) and a longer median symptom duration (30 days in AK vs. 7 days all others, *P* < 0.0001).

### Microbiological Culture and Light Microscopy Results

Tables 2 and 3 summarize the organisms identified on microbiological testing in the 239 patients included in the analysis. Most patients (74%, n = 176) met the reference standard criteria of fungal positivity. These included 2 cases of mixed infection, i.e., fungal filaments detected on light microscopy but positive culture for bacteria (*Streptococcus viridans* and *Streptococcus pneumoniae,* respectively). Thirty participants had fungal filaments detected on light microscopy alone (negative culture for fungus), of whom 83% (n = 25) had used antifungal therapy prior to presentation and 50% (n = 15) were deep with the stromal infiltrate involving the posterior third of the cornea. All 17 *Acanthamoeba* cases were culture positive, and 13 of these were also light microscopy positive (none was solely light microscopy positive for *Acanthamoeba*). The culture-positivity rate for any organism was high, at 76% (n = 182).

### Detection of Fungal Filaments by IVCM

**Figure 1:**
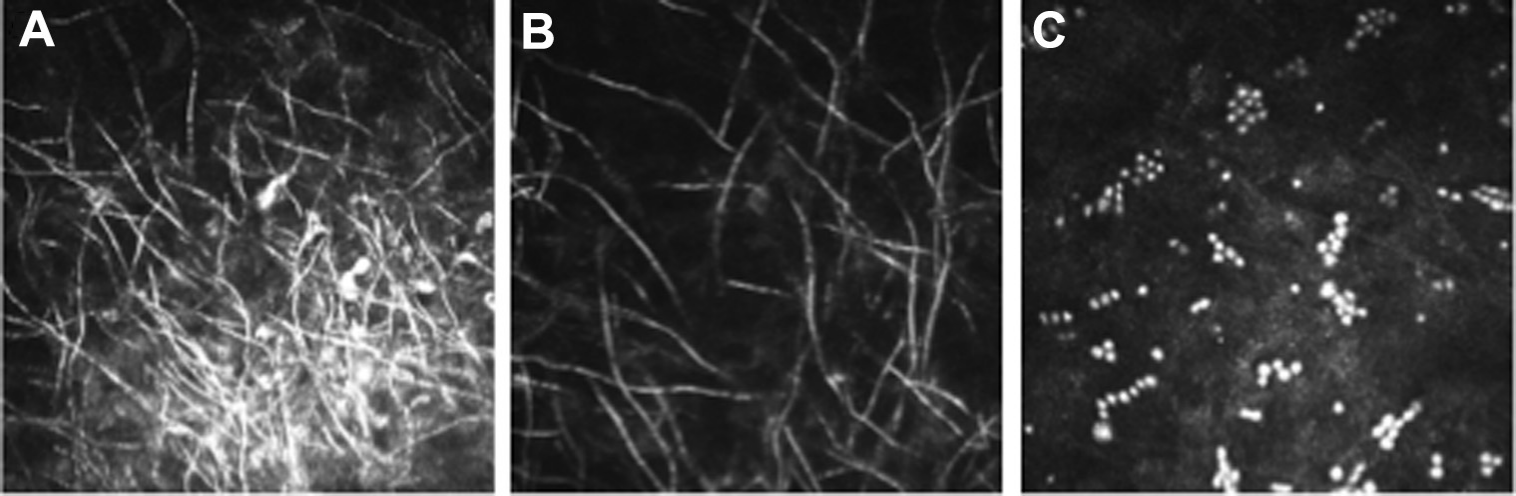
In vivo confocal microscopy (IVCM) images of *Fusarium* sp. culture-positive ulcer showing **A,** overlapping fungal filaments in the center of the ulcer and **B,** more-distinct fungal filaments at the periphery; **C,** IVCM images of an *Acanthamoeba* sp. culture-positive ulcer showing cysts in chains and clusters.

Figures 1A and B show an example of fungal filaments as seen in IVCM images of a culture-positive fungal ulcer. Overall, all 5 graders were able to definitely detect fungal filaments in the IVCM images, with a pooled sensitivity of 85.7% (95% confidence interval [CI]: 82.2%–88.6%) and pooled specificity of 81.4% (95% CI: 76.0%–85.9%), with individual grader data shown in Table 4A. Overall, the highest sensitivity (89.8%, 95% CI: 84.3%–93.8%) was achieved by the grader with access to the ulcer clinical features (grader 5). The grader with the lowest sensitivity (grader 2, 79.1%) also had the highest specificity (i.e., fewest false-positives). For only the 4 graders who were masked to clinical features, pooled sensitivity was 84.5% (95% CI: 80.8%–87.6%) and pooled specificity was 82.0% (95% CI: 75.7%–86.9%). Cases with earlier presentation and shorter symptom duration (≤4 days) had the highest pooled sensitivity for all 5 graders, 95% (95% CI: 88–98%), but the lowest pooled specificity, 53% (95% CI: 39%–66%). As symptom duration increased to >10 days, the pooled sensitivity reduced to 72% (95% CI: 64%–78%), with a concomitant increase in sensitivity, to 91% (95% CI: 84%–95%), as shown in Table 5.

There was a strong intergrader agreement among all 5 masked graders' scores for definite fungus, with a κ score of 0.88 (*P* < 0.0001). The κ scores for intragrader agreement (i.e., test reproducibility) were between 0.88 and 0.95 (*P* < 0.0001), i.e., strong to almost perfect agreement.

IVCM images for the 3 culture-positive *Nocardia* spp. cases were classified by 4 of the 5 graders as not having filamentous structures.

### IVCM False-Positives or False-Negatives for Fungus

**Figure 2:**
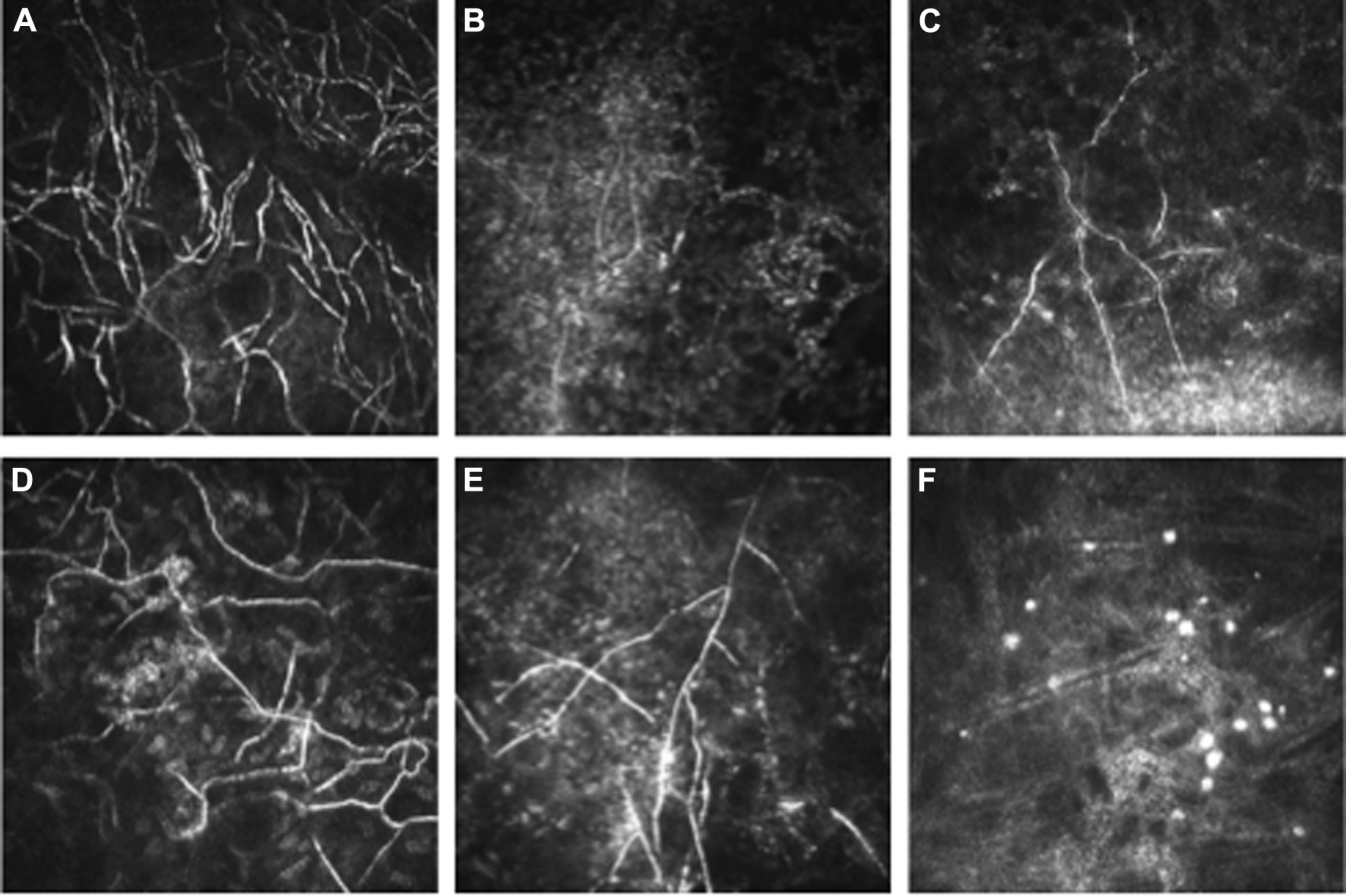
In vivo confocal microscopy images of 6 culture-negative and light microscopy-negative ulcers in which graders detected fungal filaments **A–E**, or Acanthamoeba cysts (**F**). Note the similarity of cyst appearance to those in Figure 1 image **C** with a similar absence of inflammatory cell infiltrate in the corneal stroma.

Ten patients were microbiologically negative for fungus, but ≥4 graders categorized these images as showing definite fungus (i.e., IVCM false-positives). Figure 2 shows examples of the fungal branching structures seen in these IVCM images. Of these 10 ulcers, 9 were noted to be deep with extension into the posterior third of the cornea on slit lamp examination or IVCM imaging.

Conversely, 9 patients were microbiologically positive for fungus but graded by all 5 graders as having no fungal filaments on IVCM (i.e., IVCM false-negatives). On further IVCM imaging ≤21 days after presentation, fungal filaments were still not detected in 5 patients and the remaining 4 patients had progressive corneal thinning or perforation that prevented further IVCM imaging from being performed. Five patients had surface plaques at presentation that caused high reflectivity and difficulty in imaging the ulcer clearly using IVCM. The spectrum of organisms grown from the IVCM false-negative ulcers included *Fusarium* sp. (n = 4), *Aspergillus* sp. (n = 3), *Cylindrocarpon* sp. (n = 1); in 1 patient, no organism was grown but fungal filaments were detected in corneal scrapings on light microscopy.

### IVCM Detection of *Acanthamoeba* Cysts

For definite detection of *Acanthamoeba* cysts, the 5 graders had a pooled sensitivity of 88.2% (95% CI: 76.2%–94.6%) and pooled specificity of 98.1% (95% CI: 94.9%–99.3%). The 4 graders masked to clinical features had a very similar pooled sensitivity, 88.5% (95% CI: 73.0%–95.6%), and pooled specificity, 98.0% (95% CI: 93.3%–99.4%). The grader with access to clinical-feature data had a sensitivity of 88.2% and specificity of 98.6% (grader 5, Table 4B). In ulcers presenting earlier (i.e., <20 days symptom duration), the pooled sensitivity and specificity (all 5 graders) were 82% (95% CI:34%–98%) and 98% (95% CI: 95–99%), respectively. This high sensitivity and specificity was maintained in ulcers with symptom duration >30 days (Table 5).

For all 5 graders, there was a moderate intergrader agreement, with a κ score of 0.72 (*P* < 0.0001). The κ scores for intragrader agreement for definite *Acanthamoeba* cases ranged from 0.63 to 0.90 (*P* < 0.0001). *Acanthamoeba* cyst morphology is shown in Figure 1C.

### IVCM False-Positives or False-Negatives for *Acanthamoeba*

In the 1 IVCM false-positive case, culture and light microscopy results were both negative for *Acanthamoeba*, but all 5 graders detected *Acanthamoeba* cysts on IVCM. Figure 2F shows images from this patient highlighting the presence of *Acanthamoeba* cystlike structures.

There was 1 IVCM false-negative ulcer, i.e., microbiologically positive for *Acanthamoeba* sp. but no definite *Acanthamoeba* detected by any grader. Of note, 2 of the 5 graders classified the images for this ulcer as possible *Acanthamoeba*.

### Possible Fungus or *Acanthamoeba* on IVCM

In total, 71 ulcers were classified as possible fungus present by any grader, with agreement from ≥3 graders on this diagnosis in 7 of these ulcers. The reference standard was fungal positive in 75.3% (n = 55) of those graded as possible fungus. The remainder either had no growth with no organism on light microscopy (n = 9) or were culture or light microscopy positive for *Acanthamoeba* sp. (n = 3), *Nocardia* sp. (n = 2), or *S. pneumoniae* (n = 2).

For those classified as possible *Acanthamoeba* by any grader (n = 75 ulcers), only 9.3% were microbiologically positive for *Acanthamoeba* sp. (n = 7), the remainder being microbiologically positive for fungus (n = 43) or bacteria (n = 13), or with no organism detectable on culture or light microscopy (n = 12). Three or more graders were in agreement of the possible *Acanthamoeba* diagnosis in 13 ulcers, of which only 2 were *Acanthamoeba* positive using the reference standard.

At regrading, ≤57% of all images initially classified by any grader as possible fungus were shifted to the definite fungus category (n = 34/60), and 85% of these were positive for fungus according to the reference standard (n = 29/34). Of the images initially graded as possible *Acanthamoeba*, 9% (n = 8/88) were shifted to the definite *Acanthamoeba* grade at regrading, with 75% (n = 6/8) of these being microbiologically positive for *Acanthamoeba*. Very few images were converted by any grader from definite fungus to possible fungus (n = 11/438). Of these images, 6 were converted by at least 2 of the 3 graders (*Curvularia* sp. n = 2; *Fusarium* sp. n = 2; culture/light microscopy negative, n = 2), and the remaining images were culture positive for *Aspergillus flavus* (n = 2), *Fusarium* sp. (n = 1), *Nocardia* sp. (n = 1), or culture/light microscopy negative (n = 1). For *Acanthamoeba*, again few images were regraded from definite to possible (n = 9/58), with 8 images converted by at least 2 of 3 graders (4 culture positive for *Acanthamoeba* sp., 2 for *Fusarium* sp., 2 for *Nocardia* sp.), and the remaining 1 culture positive for *Fusarium* sp.

## Discussion

Large corneal ulcers can present a major diagnostic challenge, especially as they often have mixed or atypical clinical features and may be culture negative. Delays in treatment of fungal keratitis and AK in particular can lead to significant visual loss, and even loss of the eye.^5^, ^24^, ^25^ IVCM is a noninvasive method through which fungal filaments and *Acanthamoeba* cysts can be immediately detected in the patient's cornea,^9^ allowing the clinician to promptly start the correct antimicrobial therapy. In 2004, the American Academy of Ophthalmology conducted an evidence-based assessment of the value of IVCM as a diagnostic tool for MK. With only level II and III evidence available at that time, they concluded that IVCM could be useful as an adjunctive test in the diagnosis of fungal keratitis, but for AK there was sufficient evidence to support the use of IVCM as the sole diagnostic test.^26^ Since then, 2 prospective studies using the ConfoScan IVCM have found a high sensitivity and specificity for the detection of fungal filaments and *Acanthamoeba* cysts.^11^, ^12^ To the best of our knowledge, this is the first report of the high diagnostic accuracy of the HRT3 confocal microscope in the detection of fungi and *Acanthamoeba* in moderate to severe MK in a clinical setting, comparable to the results found in these previous 2 studies. The use of a multigrader approach allowed for a more accurate assessment of sensitivity and specificity. Our study demonstrated a slightly higher sensitivity for detection of *Acanthamoeba* than fungal filaments compared with that found in the study by Vaddavalli et al. We were able to study only a small number of participants with AK, and so further research is required with a larger study population, as well as with patients in earlier stages of disease, to more fully evaluate the use of HRT3 IVCM for the diagnosis of AK.

We found that experienced IVCM graders were able to detect fungi or *Acanthamoeba* in 94.8% of all culture-positive or light microscopy-positive ulcers. The main cause of IVCM false-negatives was technical difficulty in being able to obtain adequate IVCM images. Ulcers with superficial plaques caused a high level of surface reflectivity in the IVCM images, thus inhibiting recognition of fungal filaments in the ulcer surface or margins, as we found in 5 of our 9 IVCM false-negative fungal ulcers. Some patients were able to tolerate IVCM imaging for only a short time period and so only a limited number of images were obtained, and these images may not have captured pathogens present in deeper aspects of the ulcer. False-negatives due to poor patient cooperation have been previously reported with this imaging modality.^27^ In the case of our 11 IVCM false-negatives, the clinical features as well as microbiological results in these patients were able to guide appropriate treatment. Other reasons for IVCM false-negatives include the learning curve for the IVCM operator in adequately scanning the entire ulcer to capture any pathogen in the images, as well as the presence of a high degree of stromal inflammation that could mask the presence of the pathogen (i.e., through high reflectivity that reduces image contrast as with surface plaques or difficulty in identifying *Acanthamoeba* cysts in the presence of many white cells because they both have similar morphology).

We found that IVCM graders were able to detect a pathogen in 11 culture-negative and light microscopy–negative ulcers. The IVCM images in these ulcers had classical features of fungal hyphae or *Acanthamoeba* cysts, and so we believe these represent true cases of disease. In most patients, these ulcers were deep, involving the posterior third of the cornea and therefore making it less likely that superficial corneal scraping would collect viable fungi to grow in culture or to be seen via light microscopy. In such cases, IVCM is an invaluable tool to rapidly detect fungal filaments in the deep stroma, and it allows the correct antimicrobial treatment to be commenced without the need for an invasive corneal biopsy to identify the pathogen.^28^ Other causes of a false-positive IVCM for fungus include the presence of other linear branching structures such as corneal nerves and *Nocardia* sp. filaments.^29^ Only 1 grader out of 5 classified images from *Nocardia* keratitis as containing fungal filaments in this study. Because *Nocardia* sp. filaments are thinner in diameter than filamentous fungi (<1.5 microns vs. 3-6 microns, respectively),^30^ they can be more difficult to detect on IVCM images, particularly in the presence of significant stromal edema or inflammation, as in moderate to severe keratitis, but they were readily detected microbiologically in our study.

In the clinical setting, an uncertain IVCM test result can cause concern with regard to which antimicrobial therapy to commence. On further analysis of all images graded as showing the possible presence of a pathogen, 75% of those graded as possible fungus were appropriately classified when compared with the reference standard, but <10% of the images graded as possible *Acanthamoeba* corresponded to microbiologically confirmed acanthamoebal ulcers. This finding confirms the importance of adding clinical examination and microbiological testing to IVCM imaging to reach a definite diagnosis for acanthamoebal infection in particular, rather than using 1 diagnostic tool alone, as also found by other investigators.^18^

There was an apparent improvement in the certainty of diagnosis on regrading images. This learning effect was also detected by Hau et al, who found that the specificity improved for all graders on IVCM MK image regrading at a later date.^13^ They also found that, as the level of IVCM experience of the grader increased,^13^ the diagnostic accuracy for detection of MK also improved, thus indicating the importance of training in IVCM image recognition for all new graders. The IVCM grader may also benefit from having access to a clinical image of the ulcer,^18^ because our grader with access to clinical-feature information had a higher sensitivity for fungal detection.

In this study, although the graders were from a variety of backgrounds (ophthalmic nurses, optometrists, and ophthalmologists) and levels of experience, they had a high intergrader agreement for pathogen detection. We found higher κ scores for intergrader agreement than Vaddavalli et al found,^12^ a difference that may be due to the higher resolution of the HRT3 imaging system allowing for higher-definition images of the pathogen, as well as the training or experience of our confocal graders with this high-resolution imaging system. Intraobserver agreement in our study was also high, and it was better for fungal detection, with the best agreement in the most experienced observer.

Limitations of this study include the dominance of filamentary fungal keratitis and the relatively low proportion of bacterial infections. We were unable to study confocal appearances of candida keratitis, which is more common in more temperate climates. We studied only 17 cases of *Acanthamoeba*, and so further research is needed to more fully elucidate acanthamoebal detectability on IVCM imaging in a larger study. The cost of the confocal microscope may be too high for its routine uptake in areas with the highest endemicity for fungal keratitis and AK, in low- and middle-income countries in tropical regions; however, delay in treatment may result in a greater cost in the long-term because of poorer visual outcome related to delayed diagnosis.

There was a high culture-positive rate in this study. We believe there are a number of reasons for this, in addition to our inclusion of mainly larger ulcers. First, we used a microbiology service that is particularly optimized for ocular microbiology. Second, a culture could be initiated with very little delay after sample collection because the laboratory is situated next to the Cornea Clinic at Aravind Eye Hospital. Third, the standard practice is to use a Kimura spatula, which we also believe gives a more ample sample than using a needle does, thereby improving the organism detection rate. In regions with lower culture-positivity rates, the value of IVCM may be greater, as a higher proportion of cases will be culture negative. Although our study has focused on larger ulcers, we still found that IVCM can detect fungi with a high sensitivity in ulcers with only a few days' symptom duration. Also, for *Acanthamoeba* detection with IVCM, we found a high sensitivity and specificity for both early- and late-presenting ulcers.

In summary, we have found that experienced graders are able to detect fungal or acanthamoebal elements within HRT3 IVCM images with high sensitivity, specificity, and test reproducibility in moderate to severe keratitis. This imaging modality outperforms standard microbiological methods for deep ulcers in particular. The addition of clinical-feature data improved diagnostic accuracy. IVCM may therefore be considered an adjunctive tool, in addition to clinical examination and microbiological testing, for detection of fungi or *Acanthamoeba* in MK.

## Figures and Tables

**Table 1:** Demographic Data and Clinical Features of Study Participants

	Fungal Keratitis (74%, n = 176)∗	Acanthamoeba Keratitis (7%, n = 17)	Bacterial Keratitis (8%, n = 19)	Culture/Light Microscopy Negative (11%, n = 27)	*P* Value
Median age, years (range)	50 (19–80)	40 (23–70)	57 (19–80)	50 (22–74)	0.3166
Male gender, n (%)	116 (65.9)	10 (58.8)	11 (57.9)	16 (59.3)	0.7909
Symptom duration, median no. of days (range)	7 (1–90)	30 (4–155)	7.5 (2–20)	8 (2–60)	0.0001
Prior antibiotic use, n (%)†	112 (72.3)	14 (87.5)	13 (81.3)	14 (63.6)	0.3509
Prior antifungal use, n (%)†	89 (57.4)	10 (62.5)	7 (43.8)	13 (59.1)	0.7965
No. of patients with ring infiltrate, n (%)	52 (29.6)	15 (88.2)	10 (52.6)	7 (25.9)	0.0001

∗ Mixed infections included culture positive for bacteria but microscopy positive for fungus, n = 2.

† For prior drug use, n = 209 (data not available for 30 patients).

**Table 2:** Distribution of Organisms Identified by Culture or Light Microscopy

	N	%
Culture positive (n = 182)		
Acanthamoeba	17	7.1
Fungi	144	60.3
Bacteria	19	9.6
Mixed: Culture positive for bacteria, microscopy positive for fungi	2	0.8
Culture negative (n = 57)		
Culture negative but light microscopy positive for fungus	30	12.6
Culture negative but light microscopy positive for bacteria	4	1.7
Culture negative and light microscopy negative	23	9.6
Total	239	100

**Table 3:** Species Cultured for Fungi (n = 144) and Bacteria (n = 21)

Organism	Species	N	%
Fungi: hyaline	*Fusarium* sp.	73	50.7
	*Aspergillus flavus*	26	18.1
	*Aspergillus fumigatus*	5	3.5
	*Aspergillus terreus*	2	1.4
	*Cylindrocarpon* sp.	1	0.7
	Unidentified hyaline fungi	14	9.7
Fungi: dematiaceous	*Curvularia* sp.	5	3.5
	*Exserohilum* sp.	4	2.8
	*Lasiodiplodia* sp.	2	1.4
	*Bipolaris* sp.	1	0.7
	Unidentified dematiaceous fungi	11	7.6
Bacteria: gram-positive	*Streptococcus pneumoniae*	10	47.6
	*Streptococcus viridans*	3	14.3
	*Staphylococcus epidermidis*	2	9.5
	*Nocardia* sp.	3	14.3
Bacteria: gram-negative	*Pseudomonas aeruginosa*	2	9.5
	*Aeromonas* sp.	1	4.8

**Table 4A:** Sensitivity, Specificity, Positive Predictive Value (PPV), and Negative Predictive Value (NPV) for Definite Detection of Fungi on IVCM Compared with Culture or Light Microscopy

Grader	N∗	TP	TN	FP	FN	Sensitivity (%, 95% CI)	Specificity (%, 95% CI)	PPV (%, 95% CI)	NPV (%, 95% CI)
1	219	139	49	9	22	86.3 (80–91.2)	84.5 (72.6–92.7)	93.9 (88.8–97.2)	69.0 (56.9–79.5)
2	217	121	55	9	32	79.1 (71.8–85.2)	85.9 (75.0–93.4)	93.1 (87.3–96.8)	63.2 (52.2–73.3)
3	190	117	44	9	20	85.4 (78.4–90.8)	83.0 (70.2–91.9)	92.9 (86.9–96.7)	68.8 (55.9–79.8)
4	224	145	42	15	22	86.8 (80.7–91.6)	73.7 (60.3–84.5)	90.6 (85.0–94.7)	65.6 (52.7–77.1)
5†	239	158	50	13	18	89.8 (84.3–93.8)	79.4 (67.3–88.5)	92.4 (87.4–95.9)	73.5 (61.4–83.5)

FN = false-negative; FP = false-positive; TN = true-negative; TP = true-positive.

∗ The total no. of patients classified as having possible fungus by each grader and therefore excluded from this analysis are as follows: grader 1 (n = 21), grader 2 (n = 23), grader 3 (n = 49), grader 4 (n = 16), grader 5 (n = 1).

† Grader 5 was unmasked to ulcer clinical features.

**Table 4B:** Sensitivity, Specificity, Positive Predictive Value (PPV), and Negative Predictive Value (NPV) for Definite Detection of *Acanthamoeba* on IVCM Compared with That of Culture or Light Microscopy

Grader	N∗	TP	TN	FP	FN	Sensitivity (%)	Specificity (%)	PPV (%)	NPV (%)
1	208	11	187	9	1	91.7 (61.5–99.8)	95.4 (91.5–97.9)	55.0 (31.5–76.9)	99.5 (97.1–100)
2	202	12	188	1	1	92.3 (64.0–99.8)	99.5 (97.1–100)	92.3 (64.0–99.8)	99.5 (97.1–100)
3	205	12	191	1	1	92.3 (64.0–99.8)	99.5 (97.1–100)	92.3 (64.0–99.8)	99.5 (97.1–100)
4	218	12	188	14	4	75.0 (47.6–92.7)	93.1 (88.6–96.2)	46.2 (26.6–66.6)	97.9 (94.8–99.4)
5†	239	15	219	3	2	88.2 (63.6–98.5)	98.6 (96.1–99.7)	83.3 (58.6–96.4)	99.1 (96.8–99.9)

FN = false-negative; FP = false-positive; TN = true-negative; TP = true-positive.

∗ The total number of patients classified as having possible *Acanthamoeba* by each grader and therefore excluded from this analysis are as follows: grader 1 (n = 31), grader 2 (n = 37), grader 3 (n = 32), grader 4 (n = 21); 2 patients excluded by grader 3 as having ungradeable images.

† Grader 5 was unmasked to ulcer clinical features.

**Table 5:** Pooled Sensitivity and Specificity for All 5 Graders by Symptom Duration (Split by Quartile)

Organism	Symptom Duration	Sensitivity (%)	Specificity (%)
Fungal keratitis	Q1: ≤4 days	95 (88–98)	53 (39–66)
	Q2: 5–7 days	86 (81–90)	75 (64–84)
	Q3: 8–10 days	91 (85–95)	96 (84–99)
	Q4: >10 days	72 (64–78)	91 (84–95)
*Acanthamoeba* keratitis	Q1: <20 days	82 (34–98)	98 (95–99)
	Q2: 20–30 days	98 (53–100)	96 (76–100)
	Q3&4: >30 days	83 (68–92)	96 (76–99)
