# Supplementary figures and images for: Prospective Study of the Diagnostic Accuracy of the In Vivo Laser Scanning Confocal Microscope for Severe Microbial Keratitis
Source: Ophthalmology. 2016 Nov;123(11):2285–93. doi: 10.1016/j.ophtha.2016.07.009 (PMC5081072; doi:10.1016/j.ophtha.2016.07.009)

Supplementary Figure: Flow of Participants through the Study (STARD diagram)

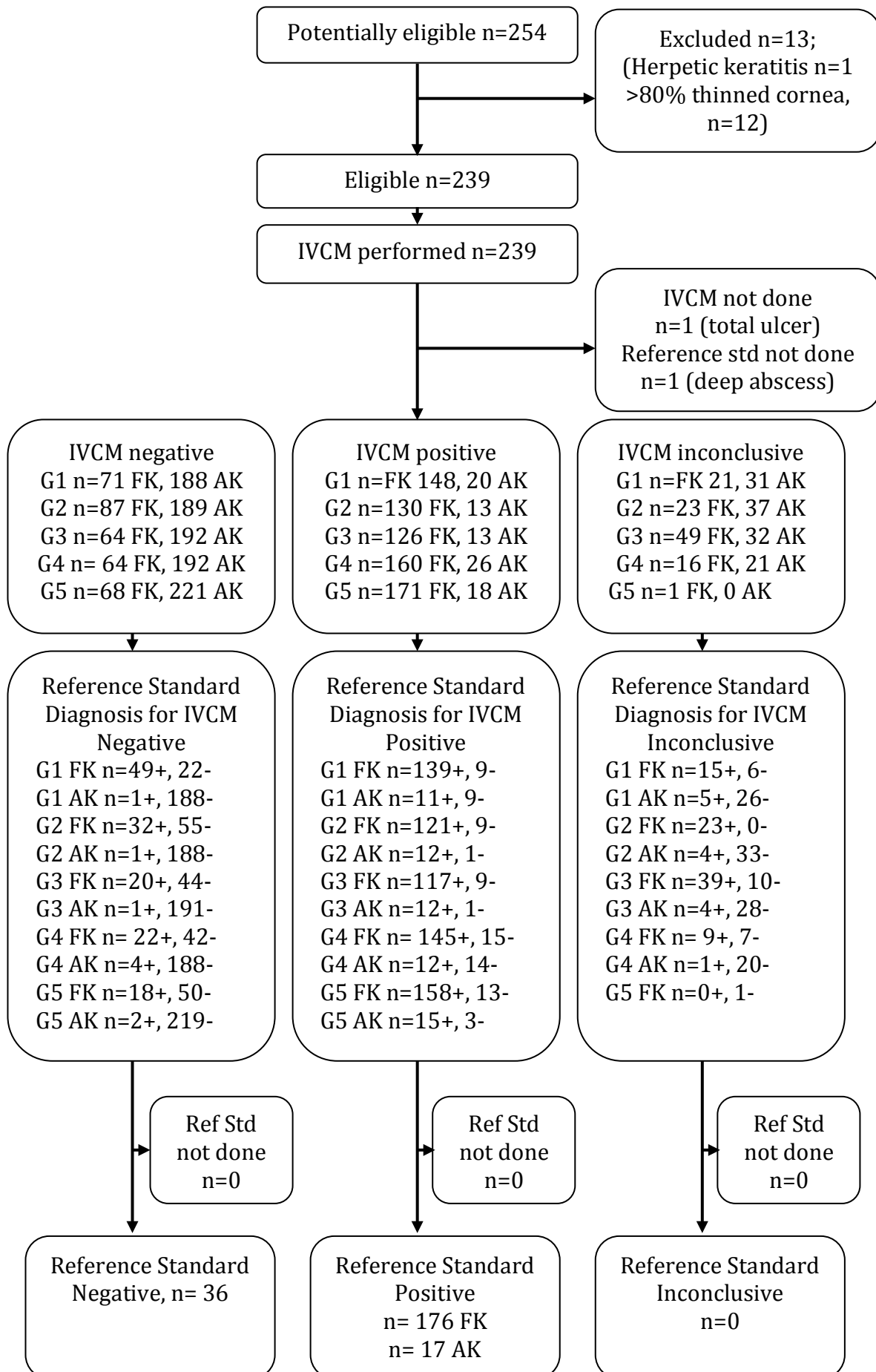

Supplement: Supplementary Figure S1 [file mmc1.pdf]
